# Full-Term Delivery and Complete Lung Recovery following VV ECMO Support Midpregnancy in a Patient with COVID-19 ARDS

**DOI:** 10.1155/2023/3472718

**Published:** 2023-03-06

**Authors:** Shelley Leong, Guillermo Moreno, Mohamed Fayed, Crystal Ives Tallman

**Affiliations:** ^1^University of California San Francisco, Fresno, USA; ^2^Department of Pulmonary and Critical Care Medicine, University of California San Francisco, Fresno, USA; ^3^Department of Obstetrics, University of California San Francisco, Fresno, USA

## Abstract

Pregnant women are especially vulnerable to coronavirus disease 2019 (COVID-19). We present a twin pregnancy case with acute respiratory distress syndrome following COVID-19 infection at 19 weeks. The patient's ARDS was successfully managed with veno-venous extracorporeal membrane oxygenation (VV ECMO). She recovered completely and delivered healthy twins.

## 1. Introduction

Maternal immunologic adaptations to pregnancy are known to increase the risk of severe respiratory disease from viral infections. Pregnancy is a risk factor for severe COVID-19 [1–4]. In one Italian study, one out of five pregnant women admitted with COVID-19 was delivered urgently for respiratory compromise or was admitted to the ICU [1]. After adjusting for other risk factors, pregnant women with COVID-19 are three times more likely to require mechanical ventilation than nonpregnant women [4]. Veno-venous extracorporeal membrane oxygenation (VV ECMO) is a rescue strategy for severe ARDS that has been used successfully in pregnant women during the COVID-19 Pandemic [5–10]. This support is not undertaken lightly, however, as ECMO support is associated with increased complications, including moderate to severe bleeding and intracranial hemorrhage [11].

We present a case of severe ARDS secondary to COVID-19 requiring VV ECMO during midpregnancy. To our knowledge, this is the first case of VV ECMO support during twin gestation for this indication, with complete recovery of lung function.

## 2. Case Presentation

A 39-year-old woman with twin pregnancies presented at 19 weeks two days of gestation with worsening shortness of breath. She tested positive for COVID-19 a week before the presentation. She reported a productive cough and loss of appetite for one week and was admitted for acute hypoxia with 82% oxygen saturation on room air. She was evaluated the day before in the emergency department for her symptoms and advised admission.

On admission, she was placed on two litres oxygen via nasal cannula. On day three, she alternated between noninvasive positive airway pressure and high-flow nasal cannula support. Due to ongoing oxygen demand, she was transferred to the ICU for further monitoring. She was treated with dexamethasone (6 mg daily), remdesivir (200 mg once, then 100 mg daily), one dose intravenous methylprednisolone (80 mg), and vitamins C, D, and Zinc.

The patient developed severe ARDS in the ICU and was then transferred to an ECMO centre for further evaluation. Her lung condition deteriorated with worsening hypoxia, and she was intubated on day six. The perinatology team evaluated her at the ECMO centre. A bedside ultrasound confirmed normally developing twins.

**Figure 1:**
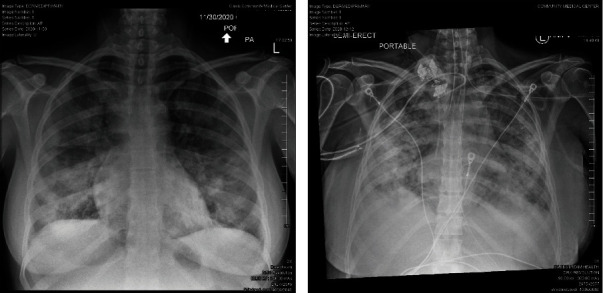
(a) Bilateral infiltrates on initial portable chest X-ray at her first ED visit. (b) Progression of bilateral infiltrates on portable chest X-ray on hospital day 12.

She remained intubated with fluctuating FiO2 support until day 13. On day 13, her ventilatory and sedative demands were significantly increasing. A chest X-ray confirmed the progression of her lung disease (Figure 1). She did not respond to prone therapy. Her tidal volumes increased to greater than 7 mL/kg, and peak ventilatory pressure the next day was 36 cmH2O. With the loss of lung protective ventilation and her rapid clinical decline, she underwent VV ECMO cannulation on hospital day 14 at 21 weeks and 1-day gestation.

A 28 Fr Crescent dual lumen catheter was placed in the right internal jugular vein. During ECMO cannulation, the patient received a weight-based heparin bolus and anticoagulation was maintained with a heparin drip titrated to anti-Xa levels. Following VV ECMO initiation, the patient's ventilator support was quickly weaned to extreme lung protective ventilation parameters in an effort to reduce the potential for lung damage and complications. This included even more protective parameters beyond ARDSnet protocol. By day two of VV ECMO support, ventilator settings were as follows: pressure control ventilation with tidal volumes < 6 cc/kg IBW, FiO2 of 50%, inspiratory pressure of 15, positive end-expiratory pressure of 10, and respiratory rate of 6. On day four of VV ECMO support, her ventilator settings had been weaned further, and FiO2 was 35%.

While supported by VV ECMO, daily awakening trials were performed, but her course was complicated by hyperactive delirium. Enteral quetiapine and diazepam allowed her to be calm and awake. On day eight of VV ECMO treatment, she could sit up at the edge of the bed with physical therapy. She required VV ECMO support for nine days due to refractory hypercapnia from her COVID-19 infection and was decannulated on hospital day 22. She was extubated on hospital day 24 to a high-flow nasal cannula and remained in the ICU until hospital day 28. She required four litres nasal cannula at the time of her downgrade to the medicine floor.

**Figure 2:**
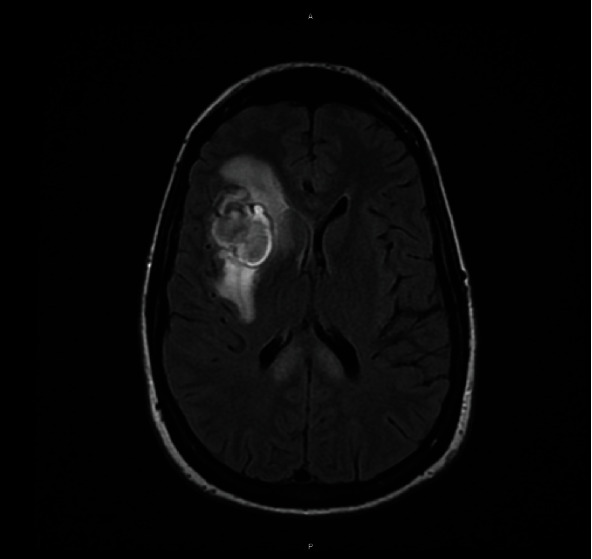
Intraparenchymal hemorrhage of the right frontal lobe on axial T2 flare MRI.

Routine fetal monitoring was initiated once she reached 23 weeks gestation. From 23 weeks to 23 weeks and six days, fetal heart rates were monitored daily. Unfortunately, the patient's hospital course was then complicated by altered mental status. An MRI of the head on day 32 confirmed a right-sided intraparenchymal hemorrhage that was 2.6 × 3.4 × 3.3 cm in size (Figure 2). The patient was transferred to the Neuro ICU for 24 hours for frequent neurologic checks. No additional bleeding or ECMO-related complications were identified.

Both twins reached a viability period of 24 weeks. Electronic fetal monitoring of both the twins was carried out until 24 + 1 weeks. Parents expressed that they would like full intervention, including cesarean delivery if needed. A biophysical profile (BPP) was performed every 2-3 days to check fetal well-being. Subsequent MRA of the head did not show any abnormalities. Other than her altered mental status, her neurologic exam was normal. The neurosurgery team recommended monitoring with a repeat MRI of the head. She was transferred back to the medicine floor the next day.

The patient was discharged to acute rehabilitation on hospital day 40. She was there for nine days before she was discharged home without any need for supplemental oxygen. Repeat head MRI showed an interval decrease of her known intraparenchymal hemorrhage. Her mental status remained normal.

The rest of her pregnancy was uncomplicated with regular twice-weekly follow up in a high-risk pregnancy clinic. She was admitted to the hospital at 37 weeks and 3 days for planned cesarean delivery and delivered healthy twins. Baby A had an uncomplicated neonatal course, spending two days in the NICU. Baby A's birthweight was 3005 g. APGARS at the time of delivery were 8 and 9 at 1 and 5 minutes, respectively. Baby B also had an uncomplicated neonatal course, spending two days in the NICU. The baby's birthweight was 2675 g, and APGARS were 8 and 8 at 1 and 5 minutes, respectively. No COVID-19 testing was done on either infant.

**Figure 3:**
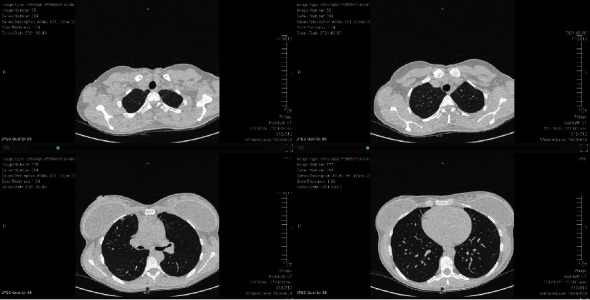
Resolution of disease seen on serial axial cuts of noncontrast chest CT six months after initial presentation.

**Figure 4:**
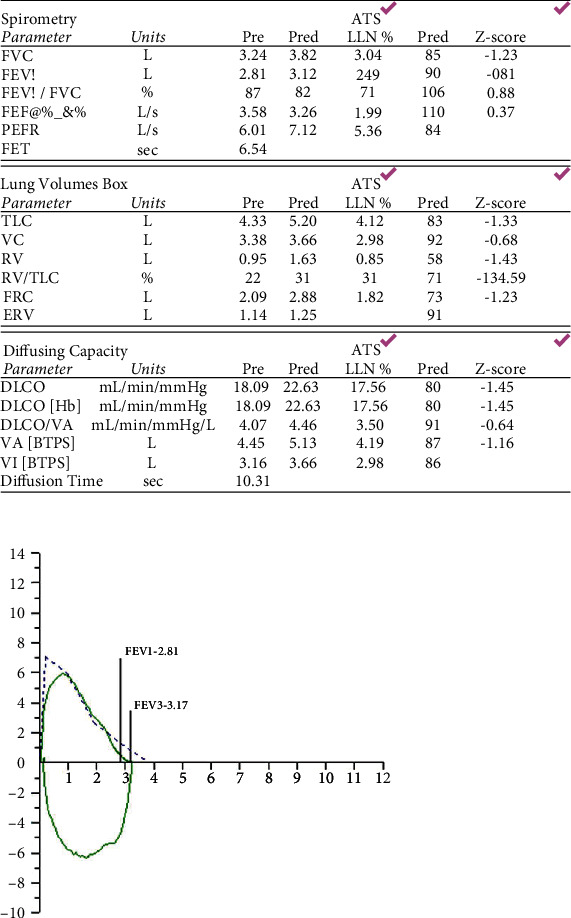
Normal spirometry, lung volumes, and diffusing capacity six months after initial presentation.

Postnatal follow-up, six months after delivery, showed complete resolution of the patient's COVID-19 disease. Her CT chest demonstrated a normal appearance of the lung parenchyma, and her pulmonary function tests had returned to normal (Figures 3 and 4).

## 3. Discussion

Pregnant women are especially vulnerable to COVID-19. Pregnant women are more likely to require intensive care and mechanical ventilation for COVID-19 ARDS than non-pregnant women [4]. The successful use of VV ECMO as a rescue therapy in pregnant women with COVID-19 ARDS has been documented in a handful of case reports [5–10]. In these case reports, these pregnant women required emergent delivery before, during, or immediately after VV ECMO support [5–10]. More recent case reports have documented successful treatment of COVID-19 ARDS with VV ECMO in pregnant women, allowing for full-term deliveries [8, 10]. Our patient is unique in that following VV ECMO support she delivered healthy, full-term twins and had complete recovery of her lung function.

Our patient had a longer hospital course compared to other case reports of pregnant women with COVID-19 ARDS treated with VV ECMO, due to other complications such as her right-sided intraparenchymal brain hemorrhage. Despite this complication, the patient had a very good outcome from a neurologic, respiratory, and pregnancy perspective. Rates of complications in pregnant women requiring ECMO support are similar to other patients requiring ECMO support. These complications include mild to moderate bleeding in 18%, severe bleeding requiring surgical intervention in 13%, and intracranial neurologic morbidity in 5% [11].

There were likely multiple factors contributing to her successful, full-term pregnancy. Early transfer to an ECMO centre with an obstetrics team familiar with high-risk pregnancies was essential to her care. Once lung protective ventilator support was no longer possible, ECMO cannulation was likely beneficial to allow her lungs to heal. Aggressive awakening trials and physical therapy supported by VV ECMO probably also added to her complete recovery.

## 4. Conclusion

A few case reports have documented VV ECMO rescue therapy in pregnant women with ARDS from COVID-19 infection. However, this is the first case demonstrating successful VV ECMO support during twin pregnancy with full-term delivery of twins. In addition, this case also demonstrates complete lung function recovery.
